# NFIX suppresses breast cancer cell proliferation by delaying mitosis through downregulation of CDK1 expression

**DOI:** 10.1038/s41420-025-02361-8

**Published:** 2025-02-25

**Authors:** Hai-Yan Ma, Rui Sun, Tian Tian, Xue-Jie Zhou, Zhao-Hui Chen, Xu-Chen Cao, Yue Yu, Xin Wang

**Affiliations:** 1 The First Department of Breast Cancer, Tianjin Medical University Cancer Institute and Hospital, National Clinical Research Center for Cancer, Tianjin, China; 2 Key Laboratory of Breast Cancer Prevention and Therapy, Tianjin Medical University, Ministry of Education, Tianjin, China; 3 Key Laboratory of Cancer Prevention and Therapy, Tianjin, China; 4 Tianjin Clinical Research Center for Cancer, Tianjin, China; 5 Department of General Surgery, Beijing Luhe Hospital, Capital Medical University, Beijing, China

**Keywords:** Cell growth, Breast cancer

## Abstract

One of the fundamental biological characteristics of malignant tumors is their uncontrolled growth and multiplication, which is a major reason why breast cancer remains incurable. The significance of NFIX in the development of various cancers has been demonstrated by an increasing number of studies in recent years. However, the role of NFIX in breast cancer has received less attention. This study investigates its expression in breast cancer and its function in inhibiting cell cycle progression. NFIX is downregulated in breast cancer compared to normal breast tissue, which impacts prognosis. In vitro and in vivo Experiments have shown that the overexpression of NIFX leads to a delay in the G2/M phase, which inhibits breast cancer cell proliferation. It thus plays a role as a tumor suppressor in breast cancer development. In terms of mechanism, upregulating NFIX causes CDK1 to be more susceptible to ubiquitination-mediated degradation. NFIX also competitively represses CDK1 transcription via YBX1. Moreover, NFIX expression in breast cancer is associated with methylation of its promoter region. Our study demonstrated that NFIX plays a critical role in CDK1-regulated cell cycle transitions and determined that NFIX inhibits cell proliferation in breast cancer.

## Introduction

Breast cancer has become one of the major cancers affecting women’s health worldwide [1]. Although treatment modalities such as surgery, chemotherapy, radiotherapy, targeted therapy, and endocrine therapy have dramatically improved the prognosis of breast cancer, many patients, particularly those with advanced stages of the disease, still face a poor prognosis [2, 3]. We still do not fully understand the molecular pathogenesis of breast cancer [4]. Therefore, an in-depth study of the molecular mechanism underlying abnormal gene expression in breast cancer progression, as well as the discovery of biomarkers and efficient therapeutic targets related to this progression, holds great scientific and clinical value for improving the survival expectancy of breast cancer patients and achieving precise treatment of the disease.

The Nuclear Factor I (NFI) gene family was initially identified based on a purified protein from human HeLa cells that plays a role in the in vitro replication of adenovirus DNA [5]. The human NFI family comprises four related factors: NFIA, NFIB, NFIC, and NFIX [6]. Over the last few decades, members of the NFI family have been shown to regulate cell proliferation and differentiation in multiple organs [7, 8]. In addition, other research indicates that the NFI gene is closely associated with the development of various types of cancer [9]. Song et al. found that NFIA shows higher expression levels in astrocytoma and is strongly associated with disease prognosis [10]. Dooley et al. demonstrated that NFIB is distinctly expressed in human small-cell lung cancer, regulating the apoptosis and proliferation of cancer cells [11]. Conversely, NFIX expression is diminished in non-small cell lung carcinoma, serving as an independent prognostic indicator of poor outcomes, although no significant differences have been observed in other lung cancer subtypes [12]. Currently, research on the role of NFIX in breast cancer remains limited, and its molecular mechanisms are not yet fully elucidated.

The cell cycle encompasses the period from the conclusion of one cell division to the termination of the subsequent division, and it is traditionally segmented into interphase and metaphase [13]. Abnormal cell division, caused by cell cycle disorders, is a hallmark of cancer [14]. Cyclin-dependent kinases (CDKs) were initially identified in the 20th century through an investigation involving yeast cells and embryonic extracts, leading to the elucidation of their mechanism [15]. CDKs are highly conserved Ser/Thr protein kinases that are crucial for the regulation of the cell cycle [16]. CDK1 is an important member of CDKs involved in controlling events such as DNA replication, mRNA transcription and translation, DNA repair, and cellular morphogenesis, and is closely associated with breast cancer development [17]. Notably, CDK1 is involved in the G2 to M phase transition and mitotic initiation of the cell cycle [18]. CDK1 forms a complex with its regulator, Cyclin B1. Upon activation of this complex, CDK1 regulates multiple substrate proteins through phosphorylation, thereby directing cells into mitosis [19]. Therefore, Identifying genes that can regulate CDK1 is essential for elucidating the cell cycle regulatory network.

In this study, we examined the role of NFIX in breast cancer progression and the underlying molecular mechanisms. NFIX exhibits decreased expression in breast cancer cells, and it impedes breast cancer progression by targeting CDK1 and triggering a G2/M cell cycle arrest. These results offer a molecular foundation for future research into novel therapeutic targets and prognostic markers.

## Results

### NFIX is downregulated in breast cancer and is related to survival

**Fig. 1:**
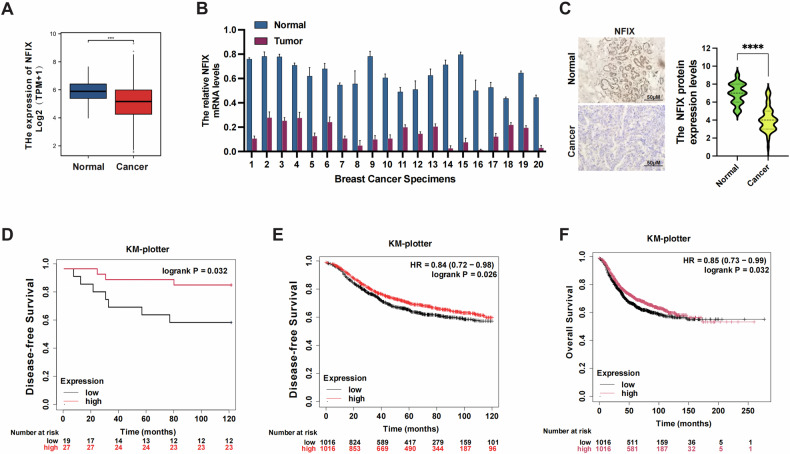
High NFIX expression predicts favorable outcomes in patients with breast cancer. **A** The NFIX expression levels in invasive breast carcinoma and normal tissues. **B** Expression of NFIX mRNA in 20 paired breast cancer tissues. **C** The protein expression levels of NFIX in breast cancer tissues and paired normal tissues according to IHC. **D** Kaplan–Meier analysis of disease-free survival in patients with different NFIX expression levels in 46 cases randomly selected from breast cancer paraffin specimens in our hospital. **E**, **F** Kaplan–Meier analysis of the disease-free survival and overall survival in patients with different NFIX mRNA expression levels in TCGA database patients with breast cancer, as determined using KM plotter. All experiments were repeated three times. Statistical significance was presented as *P < 0.05, **P < 0.01 or ***P < 0.001.

The expression levels of the NFI family in different cancers were analyzed using the TCGA database. NFI family showed abnormal expression in many cancers. Among all NFI members, the expression of NFIX was noticeably lower in breast cancer (Fig. S1). To understand the role of NFIX in breast cancer and its potential as a therapeutic target, we first examined the RNA sequencing data of breast cancer (BC) from The Cancer Genome Atlas (TCGA). We found that NFIX was significantly downregulated in breast cancer samples compared with adjacent normal samples (Fig. 1A). We then analyzed NFIX expression in 20 paired breast cancer tissues using RT-qPCR and immunohistochemistry experiments and found lower levels of NFIX expression in BC tissues compared with normal tissues, confirming the results in the database (Fig. 1B, C).

We randomly selected paraffin tissue from 46 breast cancer patients from the hospital specimen bank. These patients contain complete clinicopathological information. The expression of NFIX was detected by IHC staining, and the relationship between the expression of NIFX and the prognosis of patients was analyzed. Based on the expression levels, we categorized the patients into two expression groups: high (*n* = 19) and low (*n* = 27) NFIX expression. According to Kaplan–Meier (KM) curve analysis, patients in the high-expression NFIX group outlived those in the low-expression group in terms of disease-free survival (DFS) (*P* = 0.032, Fig. 1D). The data from breast cancer patients were then analyzed using a KM plotter (http://kmplot.com/analysis). The results indicated that patients with high NFIX expression had better DFS and overall survival (OS) compared with those in the low expression group (Fig. 1E, F). These findings suggest that NFIX is expressed at lower levels in breast cancer and high expression is associated with better DFS and OS.

### NFIX expression in breast cancer is regulated by methylation of its promoter region

**Fig. 2:**
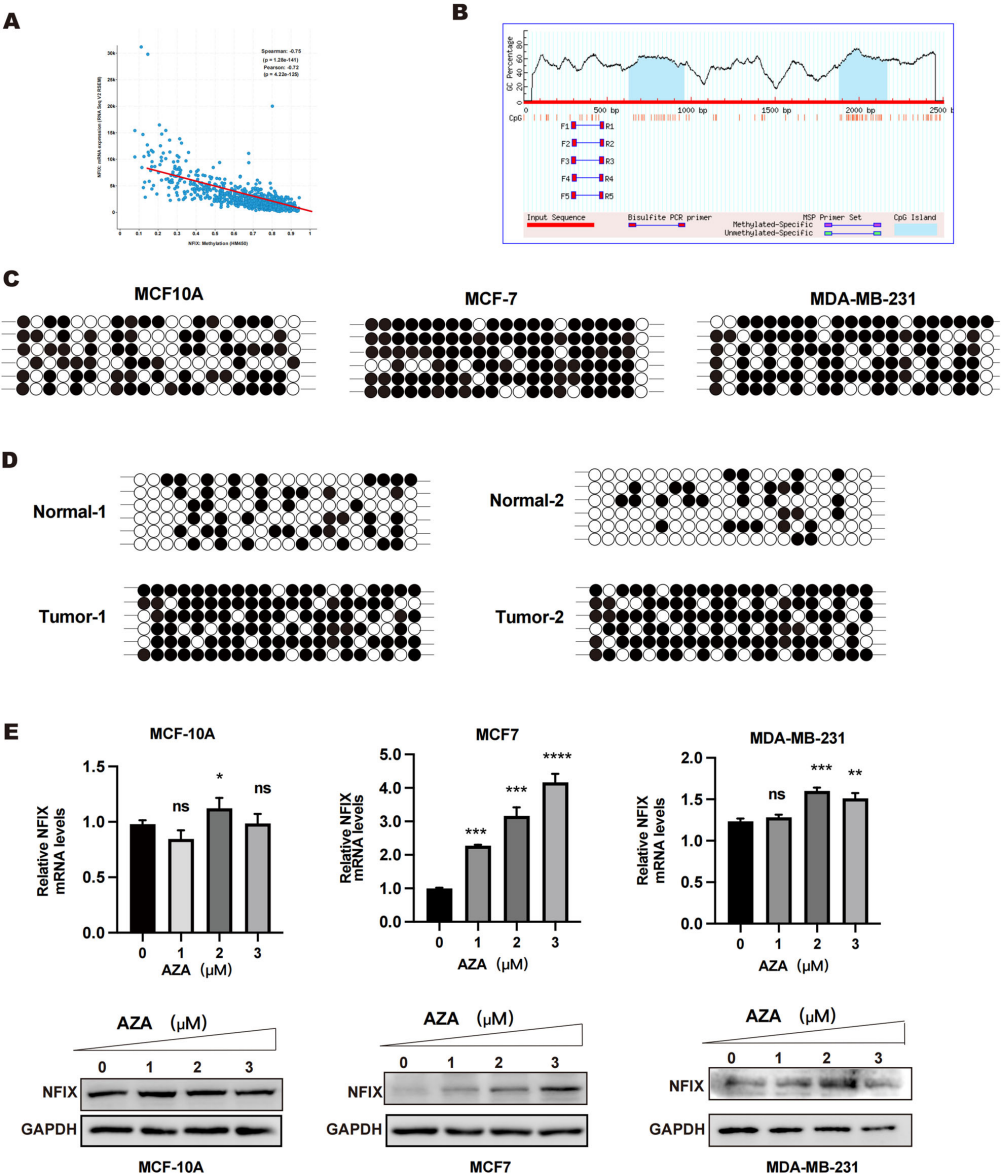
DNA methylation of NFIX in breast cancer cells. **A** The correlation between NFIX expression and DNA methylation. **B** The CpG islands in the promoter region of NFIX predicted by the Meth primer website. **C** The methylation status of NFIX detected by BSP sequencing in breast cancer cell lines. **D** The methylation status of NFIX detected by BSP sequencing in paired breast cancer tissues. **E** The NFIX expression levels detected by qRT-PCR and western blot analysis after treatment of MCF 10A cells, MCF7 cells, and MDA-MB-231 cells with AZA in a dose escalation gradient. All experiments were repeated three times. Statistical significance was presented as *p < 0.05.

Previous studies have reported a close correlation between NFIX gene expression and DNA methylation of its promoter [12]. To examine the potential relationship between DNA methylation and NFIX expression in breast cancer, we utilized cBioPortal (http://www.cbioportal.org/) to study the correlation between NFIX and the methylation status of its promoter. The findings indicated that NFIX expression and methylation regulation had the strongest association (Fig. 2A). The Meth Primer online tool (http://www.urogene.org/methprimer/) revealed that the NFIX promoter region (−1355/−1050) contains a long-range CpG island, which may be regulated by methylation (Fig. 2B). BSP sequencing was employed to determine the methylation status of the NFIX promoter in two paired cases of breast cancer tissues and breast cancer cell lines (Fig. 2C, D). The results suggest that the expression of NFIX in breast cancer may correlate with the degree of methylation of the NFIX promoter.

To confirm that NFIX expression in breast cancer is regulated by methylation, we treated normal breast epithelial cell lines and wild-type breast cancer cell lines with the DNA methyltransferase inhibitor AZA at concentrations of 0, 0.5, 1.0, 1.5, 2.0, and 2.5 µM. The levels of NFIX expression were verified by quantitative reverse transcription PCR (qRT-PCR) and immunoblotting analysis. We found higher methylation in the proximal promoter region of NFIX in MCF7 cell lines, which exhibit lower NFIX expression. In contrast, we observed, we observed lower methylation in the proximal promoter region of NFIX in the MCF10A cell line, which has higher NFIX expression. In MDA-MB-231 cells, low concentrations of AZA have no effect on the expression of NFIX, while high concentrations of AZA can also cause significant changes in NFIX expression (Fig. 2E). To summarize, the methylation level of NFIX in breast cancer increases, leading to its downregulation.

### Overexpression of NFIX inhibits breast cancer cell proliferation

**Fig. 3:**
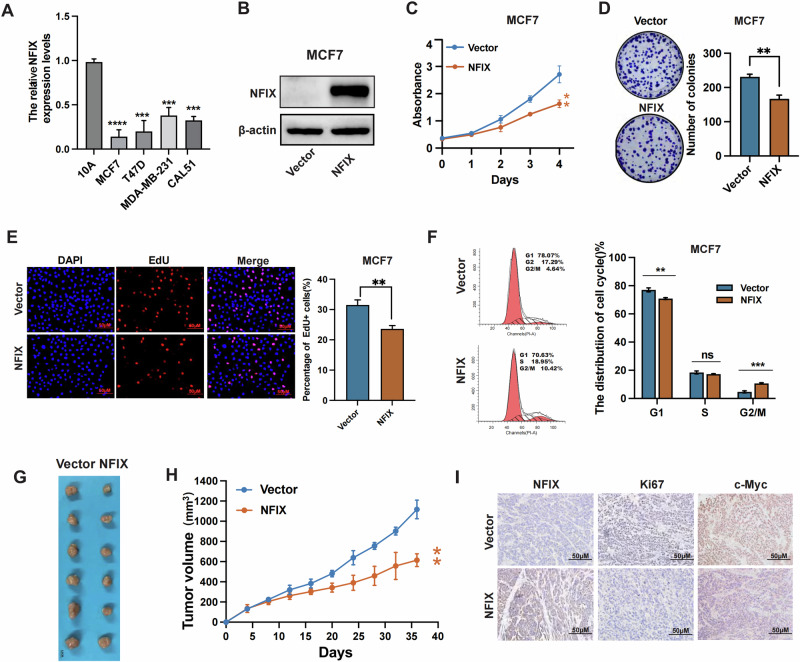
Overexpression of NFIX inhibits breast cancer cell proliferation. **A** Expression of NFIX mRNA in normal breast epithelial cells and in breast cancer cell lines. **B** NFIX expression in stable NFIX-overexpression cells (MCF7-NFIX) and control cells (MCF7-Vector) was determined by western blot analysis. **C** MTT assay of cell growth in cells treated as in (**B**). **D** Colony formation of the cells treated as in (**B**). **E** EdU assay measuring the proliferation ability of the cells described in (**B**). **F** Flow cytometry analysis of the cell cycle distribution of the cells described in (**B**). **G** The representative photo of the tumors formed by NFIX-overexpressing cells or control cells at harvest time. **H** Tumor growth curves of subcutaneous xenograft tumors comprising NFIX-overexpressing or control cells at the indicated times. **I** NFIX, Ki-67, and c-Myc protein expression levels in the xenograft tumors. All experiments were repeated three times. Statistical significance was presented as *P < 0.05, **P < 0.01 or ***P < 0.001.

To investigate the role of NFIX in breast cancer proliferation, we examined its expression of NFIX in normal breast cell lines and breast cancer cell lines. The RT-qPCR results indicated that NFIX was expressed at low levels in breast cancer, with the lowest expression observed in the MCF7 cell line compared to the other cell lines (Fig. 3A). Therefore, we infected MCF7 cells with an NFIX overexpression vector or an empty lentiviral plasmid to construct stable NFIX overexpression cell lines (MCF7-NFIX) and control cells (MCF7-Vector). Western blotting confirmed that NFIX was effectively overexpressed (Fig. 3B). Proliferation assays revealed that overexpression of NFIX could inhibit the cell viability in vitro, with consistent results obtained from the MTT assay (Fig. 3C), colony formation assay (Fig. 3D), and EdU assay (Fig. 3E). Moreover, the flow cytometry results showed that NFIX overexpression reduced the cell proliferation rate (Fig. 3F). Next, we further validated the role of NFIX in breast cancer proliferation through in vivo animal experiments. The MCF7-NFIX cells and MCF7 Vector cells were implanted into the fat pad of NSG mice, and tumor growth was recorded at regular intervals. Overexpression of NFIX in MCF7 cells significantly inhibited tumor growth in vivo (Fig. 3G, H). IHC staining revealed that the expression of Ki-67 and c-Myc was down-regulated in tumors from MCF7-NFIX mice compared to MCF7-Vector mice (Fig. 3I). To better validate this result, we transfected MDA-MB-231 and CAL51 cells with vectors expressing NFIX small hairpin RNA or negative control RNA to obtain stable NFIX-downregulated cell lines (231-shNFIX, CAL51-shNFIX) and control cells (231-shControl, CAL51-shControl). The results showed that the downregulation of NFIX promoted the growth of breast cancer cells (Fig. S2). In summary, these results indicate that overexpression of NFIX inhibited breast cancer cell proliferation in vitro and in vivo.

### NFIX induces G2/M arrest in breast cancer cells

**Fig. 4:**
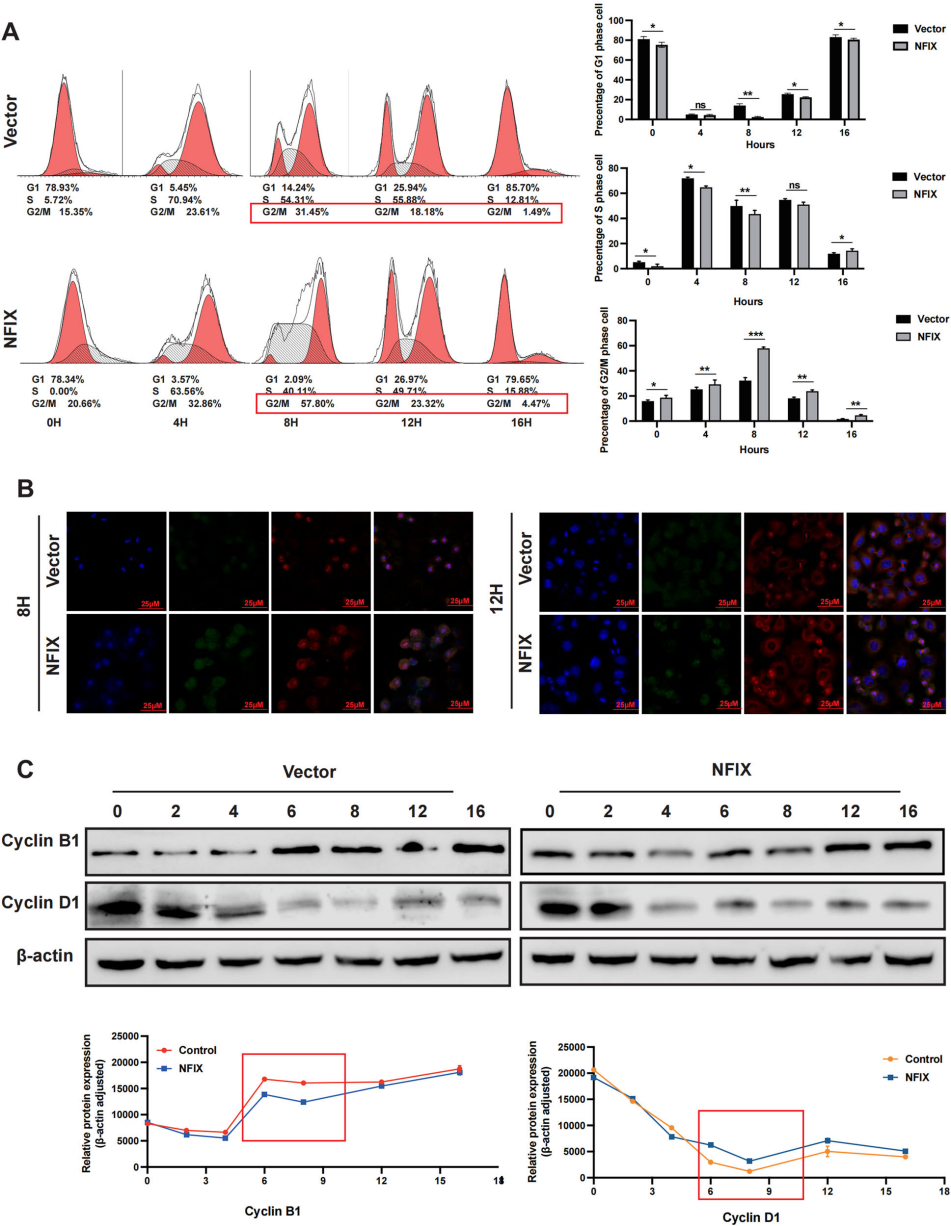
NFIX-overexpressing breast cancer cells displayed mitotic defects. **A** The cell cycle distribution of NFIX-overexpressing cells (MCF7-NFIX) and control cells (MCF7-Vector) was analyzed by flow cytometry. These cells were synchronized at the G1/S transition by a double-thymidine block and released for the indicated times. **B** Immunofluorescence staining for NFIX (green) and α-tubulin (orange), with counterstaining of nuclei using 4′,6-diamidino-2-phenylindole (DAPI, blue) in cells synchronized as in (**A**). **C** Cyclins B1 and D1 expression in cells treated as in (**A**) was detected by immunoblotting. All experiments were repeated three times.

The cell cycle experiments showed that overexpression of NFIX caused an increase in the proportion of MCF7 cells in the G2/M phase. To further investigate the functions of NFIX in the breast cancer cell cycle, we performed a cell synchronization experiment using MCF7-NFIX and MCF7-Vector cells to analyze the cell cycle transitions. We used the thymidine nocodazole cell cycle synchronization method to synchronize breast cancer cells in the G1/S phase. Cell cycle analysis was conducted at 0, 4, 8, 12, and 16 h following cell cycle release. The proportion of cells in the G2/M phase was considerably higher in the MCF7-NFIX group than in the NFIX-Vector group (57.80 vs. 31.45%, Fig. 4A). Immunofluorescence staining showed an increase in the number of metaphase cells and a decrease in the number of telophase cells among the MCF7-NFIX cells compared to MCF7-Vector cells at 12 h following cell cycle release (Figs. 4B and S3). Additionally, Cyclin D1 expression was higher than that of the control group at 4 h of release, according to western blots, while Cyclin B1 expression in the NFIX overexpression group was significantly lower than that of the control group at 6–8 h of release (Fig. 4C). In summary, these results suggest that overexpression of NFIX leads to G2/M phase arrest in breast cancer cells.

### CDK1 is a target of NFIX

**Fig. 5:**
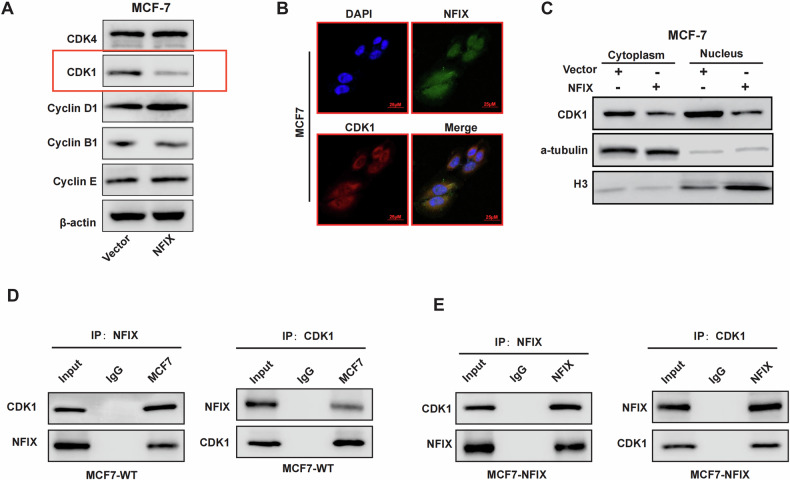
NFIX interacts with CDK1. **A** Expression of cycle-related proteins in NFIX overexpression group and control group. **B** The localization of NFIX and the intracellular colocalization between NFIX and CDK1 visualized by IF. **C** The CDK1 expression level in the nucleus and cytoplasm was detected by a nucleocytoplasmic separation western blot. **D** The interaction of NFIX and CDK1 was verified by endogenous co-immunoprecipitation. **E** The interaction of NFIX and CDK1 was verified by exogenous co-immunoprecipitation. All experiments were repeated three times.

To investigate the mechanism by which NFIX plays a role in the breast cancer cell cycle, we examined the expression of cell cycle-related protein in the MCF7-NFIX and NFIX-Vector groups using western blot experiments and found that when NFIX was overexpressed, the expression of CDK1 proteins was significantly suppressed (Fig. 5A). Next, we performed immunofluorescence experiments and confirmed that NFIX and CDK1 have co-localization in cells (Fig. 5B). To further elucidate the regulatory role of NFIX on CDK1 protein expression, we conducted nucleoplasm separation experiments to assess the impact of NFIX on the expression levels of CDK1 in both cytoplasmic and nuclear compartments. The results indicated a reduction in CDK1 levels in both the cytoplasm and nucleus in the presence of NFIX (Fig. 5C). To verify the interaction between NFIX and CDK1 proteins, we performed co-immunoprecipitation (Co-IP) assays using both endogenous proteins and exogenous overexpression of the NFIX plasmid. The funding demonstrated that CDK1 interacts with and binds to both endogenous NFIX (Fig. 5D) and exogenously expressed NFIX (Fig. 5E).

### NFIX is involved in regulating the degradation of the CDK1 ubiquitination pathway

**Fig. 6:**
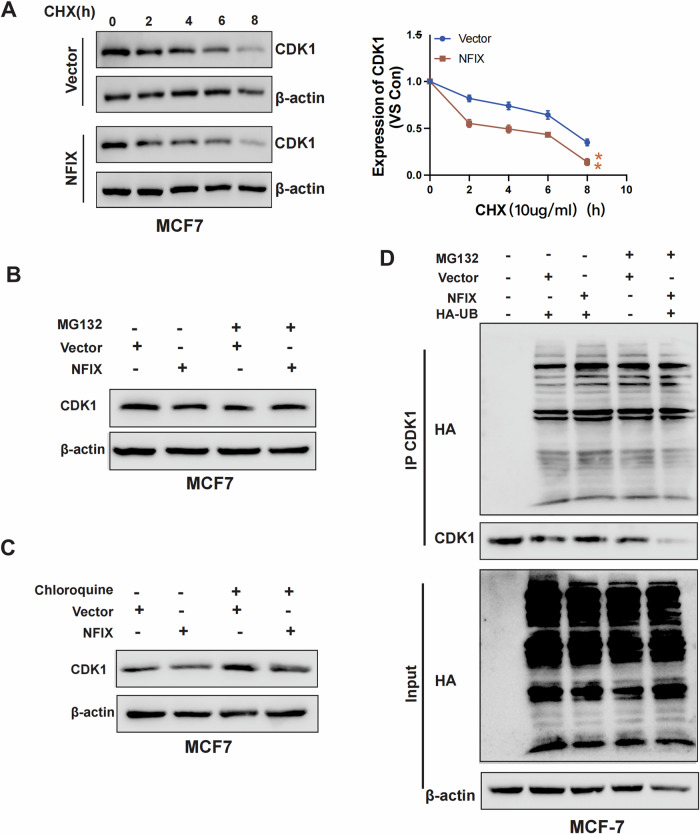
NFIX promoted the degradation of CDK1. **A** NFIX-overexpressing cells (MCF7-NFIX) and control cells (MCF7-Vector) were treated with 200 µM CHX for the indicated times. The CDK1 protein levels were analyzed by western blotting. **B** NFIX-overexpressing cells (MCF7-NFIX) and control cells (MCF7-Vector) were treated with MG132 or dimethyl sulfoxide (DMSO, i.e., vehicle) for 8 h. The cells were labeled and analyzed by western blotting. **C** Western blot detection of CDK1 expression levels in MCF7-Vector and MCF7-NFIX cells after treatment with the lysosomal inhibitor chloroquine. **D** Overexpression of NFIX promoted CDK1 ubiquitination in cultured cells. All experiments were repeated three times.

To clarify whether NFIX promotes the degradation of CDK1, we investigated the effect of NFIX on CDK1 protein stability using a cycloheximide pulse-chase experiment. As shown in Fig. 6A, the half-life of CDK1 protein was significantly shorter in MCF7-NFIX cells compared to MCF7-Vector cells, suggesting that NFIX promoted the degradation of CDK1. To clarify the pathway through which NFIX affected the degradation of CDK1, MCF7-NFIX, and MCF7-Vector cells were treated with the ubiquitination inhibitor MG132 and the lysosomal inhibitor chloroquine. The results showed that MG132 significantly upregulated CDK1 expression in MCF7-NFIX cells (Fig. 6B), while chloroquine had no effect on CDK1 expression in these cells (Fig. 6C). Further transfection with an HA-Ub expression vector followed by MG132 treatment demonstrated that NFIX overexpression significantly promoted CDK1 ubiquitination and degradation (Fig. 6D). These results confirmed that NFIX reduced the protein levels of CDK1 by promoting its ubiquitination.

### NFIX competitively inhibits CDK1 via YBX1

**Fig. 7:**
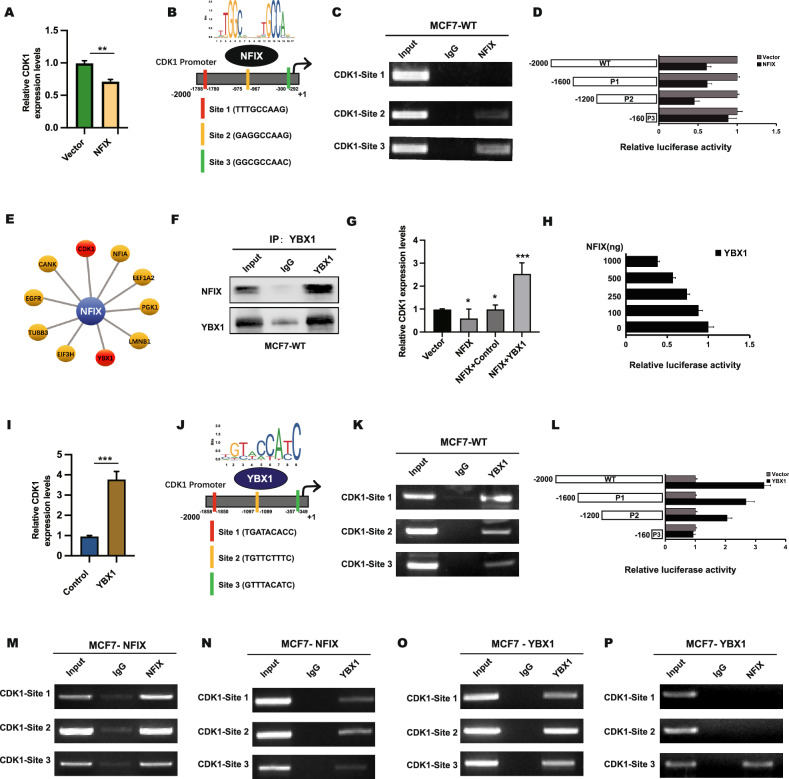
NFIX transcriptionally inhibited CDK1 through YBX1. **A** The CDK1 mRNA expression level after transfection with the NFIX expression plasmid. **B** The predicted binding sites of NFIX in the promoter region of CDK1. **C** The binding of NFIX to site1, site2, and site3 according to ChIP analysis. **D** Dual-luciferase reporter assays were used to analyze the regulation of CDK1 promoter activity by NFIX. **E** The top 10 interacting proteins identified by mass spectrometry. **F** The interaction between YBX1 and NFIX was verified using a co-IP assay. **G** The expression levels of CDK1 in NFIX-overexpressing cells with or without transfection with the YBX1 plasmid and in the corresponding control cells were determined by qRT-PCR. **H** Relationship between NFIX expression and YBX1 luciferase intensity. **I** The CDK1 expression level after transfection with the YBX1 expression plasmid. **J** The predicted binding sites of YBX1 in the promoter region of CDK1. **K** According to ChIP analysis, the binding of YBX1 to site1, site2, and site3 is in wild-type MCF7 cells. **L** Dual-luciferase reporter assays were used to analyze the regulation of CDK1 promoter activity by YBX1. **M**, **N** The binding of NFIX and YBX1 to the CDK1 promoter region after transfection of the NFIX plasmid was verified by ChIP analysis. **O**, **P** The binding of NFIX and YBX1 to the CDK1 promoter region after transfection of the YBX1 plasmid was verified by ChIP analysis. All experiments were repeated three times.

As NFIX was predicted to be a transcriptional factor, we next investigated whether NFIX could regulate CDK1 at the transcriptional level. qRT-PCR analysis revealed reduced levels of CDK1 mRNA expression in NFIX-overexpressing cell lines compared to control cells (Fig. 7A). We used the Jaspar website to predict three regions of NFIX that could potentially bind to the CDK1 promoter region (−2000~1) (Fig. 7B). The regulation of the CDK1 promoter by NFIX was verified by ChIP analysis, and the results showed that NFIX was enriched at site 2 (−1794/−1505) and site 3(−1085/−854) (Fig. 7C). A luciferase reporter assay also confirmed the binding of NFIX to the CDK1 promoter sites (Fig. 7D). Our mass spectrometry analysis also revealed that the transcription factor YBX1 binds specifically to NFIX (Fig. 7E). We next demonstrated that YBX1 can interact with NFIX through CO-IP experiments (Fig. 7F). RT-qPCR experiments demonstrated that when YBX1 was overexpressed, the ability of NFIX to inhibit CDK1 expression was diminished (Fig. 7G). A luciferase reporter assay demonstrated that the transcriptional activity of YBX1 was negatively correlated with NFIX (Fig. 7H).

Furthermore, YBX1-overexpressing cell lines exhibited higher levels of CDK1 mRNA expression than control cells, as determined by RT-qPCR analysis (Fig. 7I). The Jaspar website predicts three regions of YBX1 that could potentially bind to the CDK1 promoter region (Fig. 7J). ChIP and the luciferase reporter assays have shown that YBX1 can also bind to the CDK1 promoter region. (Fig. 7K, L). We observed that NFIX bound more strongly to the CDK1 promoter region in NFIX-overexpressing cells, whereas YBX1 binding was weaker (Fig. 7M, N). When we transfected the YBX1 plasmid in MCF7 cells, we obtained the opposite results (Fig. 7O, P), indicating that NFIX competes with YBX1 for binding to the CDK1 promoter region. Furthermore, proliferation assays and in vivo experiments similarly demonstrated that YBX1 attenuated the influence of NFIX on breast cancer cells (Fig. S4). To elucidate the interactions among CDK1, YBX1, and NFIX, we conducted overexpression studies of NFIX and YBX1 in T47D cells, followed by RT-qPCR and ChIP assays, which yielded comparable outcomes (Fig. S5).

## Discussion

As we all know, breast cancer has not yet been completely cured. Recurrence and metastasis are main challenges in its treatment, with abnormal cell proliferation being a primary factor in tumor recurrence. Previous studies have shown that abnormal expression of NFIX in tumors can activate the potential of oncogenes or tumor suppressor genes [20–22].In this study, we identified the potential role of NFIX in breast cancer, confirmed its function in vitro and in vivo, and established the potential molecular mechanism through which NFIX inhibits the breast cancer cell cycle. Specifically, we found that (i) the expression of NFIX in breast cancer is reduced, which is related to the survival rate of cancer patients; (ii) NFIX inhibits the progression of breast cancer cells both in vitro and in vivo; (iii) NFIX interacts with CDK1 and promotes its degradation through the ubiquitin-proteasome system, leading to reduced protein levels of CDK1 and thereby inhibiting the mitosis of breast cancer cells; (iv) NFIX competitively binds to the CDK1 promoter via YBX1 to inhibit CDK1 transcription; (v) the hypermethylation of the NFIX promoter negatively regulates its expression. These findings suggest that NFIX may serve as a potential biomarker and provide new insights for the treatment of breast cancer.

NFIX can inhibit the proliferation of human spermatogonia stem cells by regulating the expression of Cyclins A2, B1, and E, thus affecting DNA synthesis [23]. NFIX regulates the proliferation and DNA damage response of glioblastoma multiforme (GBM) cells through transcriptional activation of GINS1 [24]. NFIX transcriptionally upregulates Ezrin expression, thereby facilitating increased migration of GBM cells [25]. In the context of gastric cancer, NFIX influences cellular growth by modulating stemness [26]. In esophageal squamous cell carcinoma, NFIX has been shown to reduce cell proliferation and induce G1/G0 cell cycle arrest [27]. Furthermore, NFIX functions as a downstream target gene of microRNAs, impacting the development of malignant tumors [28–30]. Additionally, NFIX serves as a key regulator in lung cancer [31]. Low expression of NFIX has been reported in lung cancer, where it is associated with a poor prognosis [27]. Above all, NFIX is closely related to biogenesis, development, and cancer growth but has not been studied in breast cancer [20]. Our study reveals for the first time that NFIX expression is downregulated in breast cancer tissues and cell lines. Furthermore, patients with high NFIX expression have a better prognosis, suggesting that NFIX may be associated with breast cancer progression. Therefore, cellular functional experiments are needed next to verify the role of NFIX in breast cancer development, and further studies are required to determine the mechanisms by which NFIX acts.

The process of cell proliferation must be precisely regulated, as even the smallest alteration can lead unlimitely proliferation. This uncontrolled growth ultimately contributes to the development of malignant tumors. Therefore, understanding the mechanisms of aberrant cell division and proliferation is central to the study of breast cancer, as well as tumors in other parts of the body. We intervened in the expression of NFIX by transfecting stable overexpression plasmids or downregulation plasmids into breast cancer cell lines. We then verified the efficiency of NFIX overexpression or downregulation after lentiviral transfection using RT-qPCR and western blot experiments. The data from the MTT, clone formation, EdU, and flow cytometric cycle assays showed that, compared with their respective control groups, NFIX overexpression decreased cell proliferation ability, while NFIX downregulation caused cell proliferation ability to increase, indicating that NFIX has an inhibitory effect on cell proliferation.

The cell cycle is a central and tightly controlled process that regulates cell proliferation and development, while the loss of typical cell cycle regulation is a hallmark of malignant tumors [32]. CDK1 is a vital regulator of the cell cycle and plays an essential role in the development of cancer [33]. CDK1 is crucial in regulating mitosis, maintaining the G2/M checkpoint, initiating apoptosis, and preserving cell pluripotency and genome stability [34]. In this study, we found that NFIX may be involved in the CDK1 ubiquitination degradation pathway, and our results enriched the regulatory network related to the CDK1 protein. However, it is highly likely that NFIX, as a transcription factor, affects CDK1 degradation by regulating downstream ubiquitination-associated proteins, which in turn influence CDK1 ubiquitination. In future studies, we will further explore the important ubiquitination-related proteins that may interact with NFIX, verify the potential of NFIX to mediate CDK1 protein degradation through the ubiquitination pathway, and elucidate the mechanisms by which NFIX promotes CDK1 degradation.

The Y box binding protein 1 (YBX1) is a member of the cold shock domain protein family, which plays a variety of functions in the cytoplasm and nucleus [35]. As a transcription factor, YBX1 participates in many biological functions such as transcriptional regulation, mRNA stability, and splicing [36, 37]. Studies have shown that YBX1 can interact with NFIB as a transcription factor to regulate genes [38]. Huang et al. found that Circle NFIX can inhibit YBX1-dependent ubiquitination degradation [39]. The mass spectrometry analysis identified the genes interacting with NFIX and found that YBX1, which is also a member of the transcription factor family, could interact with NFIX. The expression of YBX1 is negatively correlated with NFIX and positively correlated with CDK1. This study demonstrated that NFIX and YBX1 are associated with CDK1 mRNA transcription and interact with each other, as shown by RT-qPCR, ChIP, and dual-luciferase reporter assay. A rescue assay further confirmed that NFIX represses CDK1 expression through YBX1.

Growing evidence shows that epigenetic regulation plays a crucial role in gene expression. A study by Jiao et al. revealed the role of methylation in regulating SEPT9 and its involvement in cervical cancer progression [40]. It was also shown that promoter hypermethylation of SEPT9 leads to its silencing [41]. Similarly, Ge et al. demonstrated that the downregulation of NFIX expression in lung cancer was related to the methylation of CpG islands in the promoter region of NFIX [12]. We predicted the CpG islands in the promoter region using the Methprimer website and found that NFIX was hypermethylated in breast cancer tissues as well as in breast cancer cell lines, as determined by BSP sequencing. Additionally, we found that the expression of NFIX in breast cancer was significantly negatively correlated with its methylation status.

## Conclusion

**Fig. 8:**
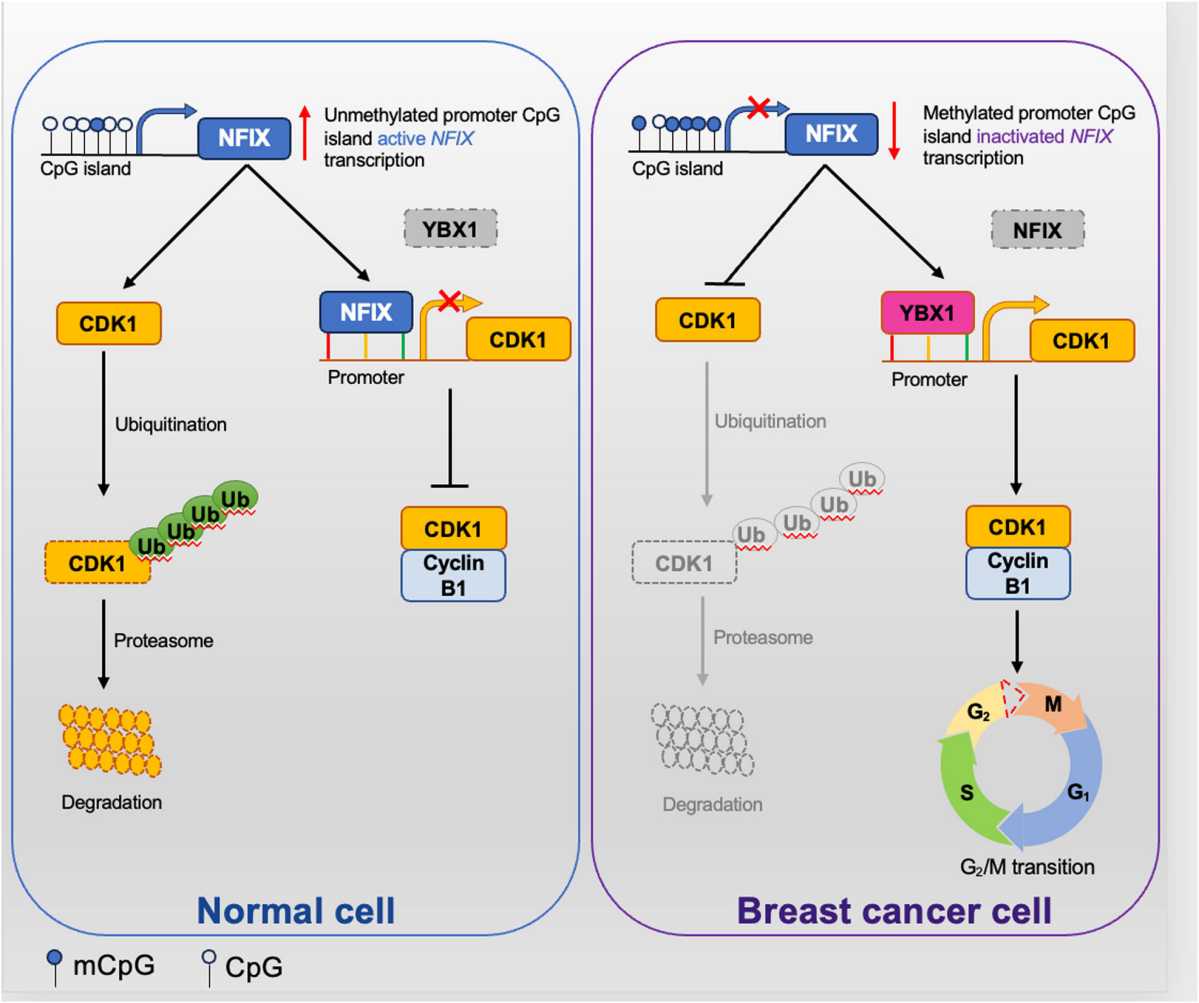
A model of the role of NFIX in breast cancer.

Our results indicate that NFIX expression is downregulated in breast cancer tissues, and overexpression suppresses cancer cell proliferation by inhibiting the G2/M phase transition of the cell cycle. NFIX acts as a transcription factor to regulate the expression of downstream genes and can also interact with target proteins to promote their ubiquitination and degradation, providing clues about the roles of NFIX in breast cancer biology (Fig. 8). Thus, NFIX is a potential biomarker and molecular target for the treatment of breast cancer.

## Materials and methods

### Cell line and cell culture

The normal human breast epithelial cell line MCF10A and human breast cancer cell lines T47D, MCF7, CAL51, and MDA-MB-231 were purchased from the Cell Bank of the Chinese Academy of Sciences (Shanghai, China). A specialized culture medium was used for MCF10A (Procell, China); DMEM medium (Gibco, USA) was used to culture MCF7 and MDA-MB-231 cells; and 1640 medium (Gibco, USA) was used to culture T47D and CAL51 cells. All media were supplemented with 1% penicillin/streptomycin solution (Gibco, Thermo Fisher Scientific) and 10% fetal bovine serum (Gibco). All cell lines were cultured in a humidified incubator with 5% CO_2_ at 37 °C.

### Clinical samples

Breast cancer tissue samples were obtained from the Cancer Hospital of Tianjin Medical University. Twenty paired breast cancer and paraneoplastic tissue samples were obtained from female patients who underwent breast cancer surgery in March 2022. Forty six randomly selected paraffin specimen samples of invasive breast cancer were obtained from female patients who underwent breast cancer surgery from January to April 2012. We also used TCGA database to analyze the expression of NFIX. This study was approved by the Institutional Review Board of TMUCIH, and all participants signed written informed consent.

### Plasmid construction and cell transfection

Full-length cDNA of human NFIX was synthesized and cloned into the expression vector (Life Technologies). To construct breast cancer cell lines with stable NFIX expression, a lentivirus-based packaging system (RiboBio, Shanghai, China) was used to prepare the virus suspension containing the NFIX overexpression plasmid, which was then used to infect MCF7 and T47D cells. Cells were transfected with NFIX-siRNAs, NFIX-shRNAs, and CDK1-stRNAs (RiboBio, Guangzhou, China) using Lipofectamine 3000 (Invitrogen, USA) according to the reagent instructions. To conduct the luciferase reporter assay of the miRNA target gene validation, sequences were synthesized and inserted into the pRL-TK vector or psiCHEK2 vector (Promega, USA). A site-directed mutagenesis kit (TransGen, China) was applied to create mutant constructs. The transfection efficiency was measured using western blotting.

### RNA isolation and qRT-PCR

Total RNA from the cultured cells was extracted using the SPARKeasy Animal Tissue/Cell RNA kit (SparkJade, China) based on the manufacturer’s instructions. A NanoDrop 2000 spectrophotometer (Thermo Scientific, USA) was used to measure the concentration and purity of RNA, after which 2 μg of RNA were reverse-transcribed into cDNA for further experiments. TransStart SYBR Green qPCR SuperMix (TransGen, China) was used to prepare the reaction system, and the quantitative qRT-PCR was conducted using the QuantStudio 5 Flex real-time PCR system (Applied Biosystems, USA). GAPDH was used as an internal standard to compare the CT values between the control and experimental groups. All samples were run in triplicate, and relative mRNA levels were normalized to the control GAPDH. All specific sequences are listed in Table S1.

### Cell proliferation assays

For the colorimetric cell viability assay (MTT assay), 1 × 10^3^ cells/well were plated into a 96-well plate and cultured in the cell incubator. Then, 10 µl of a 5 mg/ml MTT solution was added to each well at a fixed time point every day from the second to the fifth day. After further incubation for 4 h, the cells were centrifuged at 6000 × *g* for 5 min. The medium was aspirated using a needle tube, after which 150 µl DMSO was added, shaken, and mixed for 10 min, after which the absorbance at 570 nm (A_570_) of each well was measured.

For the colony-formation assay, 500 cells were inoculated into 6-well plates and cultured in a cell incubator for 3 weeks. After that, the cells were fixed with cell fixative for 30 min and stained with 0.2% crystal violet for 15 min. The stained cells were washed with PBS 3 times and photographed under a microscope to count the colonies.

EdU staining was carried out according to the instructions of the EdU detection Kit (RiboBio). The cells were seeded into 24 well plates at a density of 1 × 10^5^ cells per well. After 24 h, 1:1000 diluted EdU solution was added to the cells, followed by further culture for 2 h. The cells were then fixed with 4% formaldehyde and permeabilized with 0.5% Triton X-100 for 15 min. Then, the nuclei were labeled with EdU antibody and counter-stained with Hoechst 33258. The proportion of nuclei in the division phase was counted under the fluorescence microscope.

### Cell synchronization

Cell cycle progression was blocked by adding 2 mmol/L thymidine to the MCF7 cell culture medium and incubating for 18 h, after which the cells were cultured in the same culture medium without thymidine for 8 h and then blocked by adding 2 mmol/L thymidine and incubating for 18 h to obtain cells in S-phase Cells.

### Flow cytometry analysis

Cells were washed, digested, and resuspended for the cell cycle assay in 95% ethanol at 4 °C overnight. On the second day, the cells were washed three times with PBS and stained with PI staining solution (Cell Signaling Technology, USA) for 30 min. For the apoptosis assay, treated cells after trypsinization were stained using the Annexin V apoptosis kit (BD Pharmingen, USA). The cell cycle distribution and apoptotic rate were analyzed using a Beckman Coulter EPICS flow cytometry instrument (Krefeld, Germany).

### Western blot analysis

Cells were lysed using RIPA buffer (Solarbio, Beijing, China) with one mM PMSF, and the protein concentration was determined using the BCA Protein Assay kit (Thermo Fisher Scientific). SDS-PAGE separated proteins, transferred to a polyvinylidene fluoride membrane (Millipore, Bedford, MA, USA), incubated with the corresponding primary antibody overnight at 4 °C, and incubated with the secondary antibody at room temperature for 1 h the next day. The blots were developed using an enhanced chemiluminescence reagent (Millipore). β-actin or GAPDH was used to normalize the scale values of each individual stripe. The antibody information used in this research is listed in Table S2. The results of the quantitative WB experiment are presented in Fig. S6.

### Immunoprecipitation

A sample comprising 50 µl of the cell lysate was used as input, and the remaining cell lysate was incubated with the primary antibody at 4 °C for 2 h. Then, the antibody-target complexes were captured on protein A/protein G agarose beads (Santa Cruz, CA, USA) at 4 °C overnight. The beads were spun down at 4000 rpm for 1 min the next day, and the supernatant was discarded. The beads were washed with PBS three times and resuspended in the loading buffer. The EP tube was placed in a heating block at 100 °C for 10 min to denature the protein, and the samples were subjected to western blotting as described above.

### Immunofluorescence

A glass slide was placed on the bottom of a 24-well plate well, inoculated with 3 × 10^4^ cells, and incubated overnight in a cell culture incubator. The next day, the adherent cells were gently washed three times with PBS, fixed in 4% formaldehyde solution for 30 min, permeabilized with 0.5% Triton-X-100 in cold PBS for 15 min, washed in PBS, and then blocked with 3% fetal bovine serum protein and incubated overnight with an antibody against the target protein. The next day, the cells were washed with PBS and incubated with the appropriate fluorescence-labeled secondary antibody for 1 h. After washing with PBS, the cells were sealed with a blocker containing DAPI, and images were taken using an in-situ fluorescence microscope.

### ChIP-PCR analysis and luciferase reporter assay

According to the instructions of the CHIP kit (Millipore, USA), cells were fixed in 1% paraformaldehyde solution, lysed by adding the indicated amount of nuclear lysis solution containing protease inhibitors, and collected in clean EP tubes using a cell scraper. Chromatin was fragmented using an ultrasonic disruptor. The supernatant was centrifuged and aspirated, after which 20 μl of an antibody specific for NFIX or YBX1 and 20 μl of IgG control antibody were added and incubated overnight at 4 °C in a shaker. Then, 40 μl of protein A/G agarose beads were added to the control and experimental groups. Afterward, the antibody and chromatin complexes were separated, cross-linked, and treated to obtain purified DNA for PCR using the primers listed in Table S3. The promoter regions of CDK1 and NFIX were cloned into a pGL4.17-basic vector (Promega) and transfected into breast cancer cells along with their corresponding controls and PRL-TK plasmid (Promega). The dual-luciferase reporter assay Kit (Transgene) was utilized to detect Firefly and Renilla luciferase activities by instructions.

### Immunohistochemistry

The clinical breast cancer samples and adjacent tissues or mouse breast cancer tissues were collected and fixed with 4% paraformaldehyde at 4 °C overnight. Then, the tissues were embedded, and the wax block was cut into slices with a thickness of 4 µm using a microtome. The tissue sections were placed in an oven with a constant temperature of 60 °C overnight and dewaxed, followed by antigen retrieval and blocking of endogenous peroxidases. The corresponding antibodies were dripped onto each tissue section and incubated overnight at 4 °C. After washing with PBS, DAB chromogenic kit and hematoxylin were used for staining, ethanol, and xylene solution were used for dehydration, and neutral gum was used for sealing. The expression levels of NFIX, CDK1, Ki-67, and c-Myc were evaluated based on the product of the staining intensity score and positive cell percentage score. Intensity score: 0 points for no positive expression, 1 point for positive expression, 2 points for moderate positive expression, and 3 points for strong positive expression. Percentage score: No positive tissue expression was 0 point, 1–25% of tissue was 1 point, 26–50% of tissue was 2 points, 51–75% of tissue was 3 points, and more than 75% of tissue was 4 points.

### Xenograft

After acclimatizing female IL2 receptor ineffective (NSG) mice for 4–6 weeks, the weight baseline of the mice was measured. Then, 1 × 10^7^ tumor cells were injected subcutaneously into the mice. The size and weight of the tumors were measured every 3 days using an electronic vernier caliper. After 35 days, the mice were sacrificed, and the tumor size and weight were measured. After taking photos, the tumor was fixed for subsequent experiments. The tumor volume was calculated using the formula: tumor volume = (width **×** length)^2^/2. The experiments were approved by the TMUCIH animal ethics committee and were conducted according to the animal welfare guidelines for cancer research.

### Statistical analysis

All experiments were independently repeated three times, and the measurement data were exhibited as mean ± standard deviation. A student’s *t*-test was used to assess the statistical significance of differences between two different groups of data, and a one-way ANOVA was used to determine the importance of differences between three or more experimental groups. SPSS version 26.0 (IBM Corp., USA) was used for statistical analysis, and GraphPad Prism software version 7.0.0 (GraphPad Inc., US) was used for plotting. Differences with *P* < 0.05 were considered statistically significant.

## Supplementary information

Original western blot results

Supplementary material

## Data Availability

This article and its supplementary information files include all data generated or analyzed during this study.
