# Supplementary figures and images for: NFIX suppresses breast cancer cell proliferation by delaying mitosis through downregulation of CDK1 expression
Source: Cell Death Discov. 2025 Feb 25;11:77. doi: 10.1038/s41420-025-02361-8 (PMC11861311; doi:10.1038/s41420-025-02361-8)

Original Western Blot results


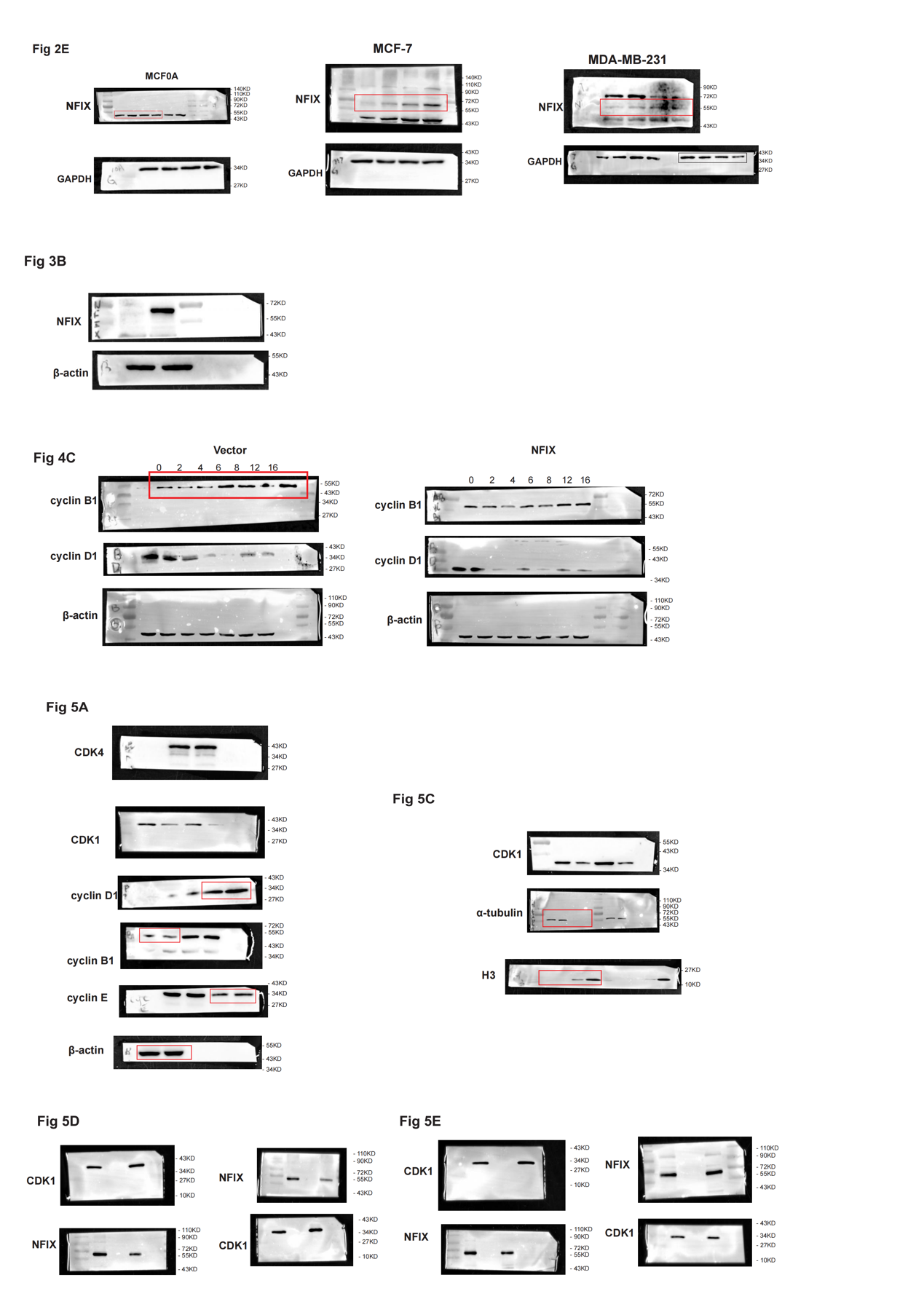


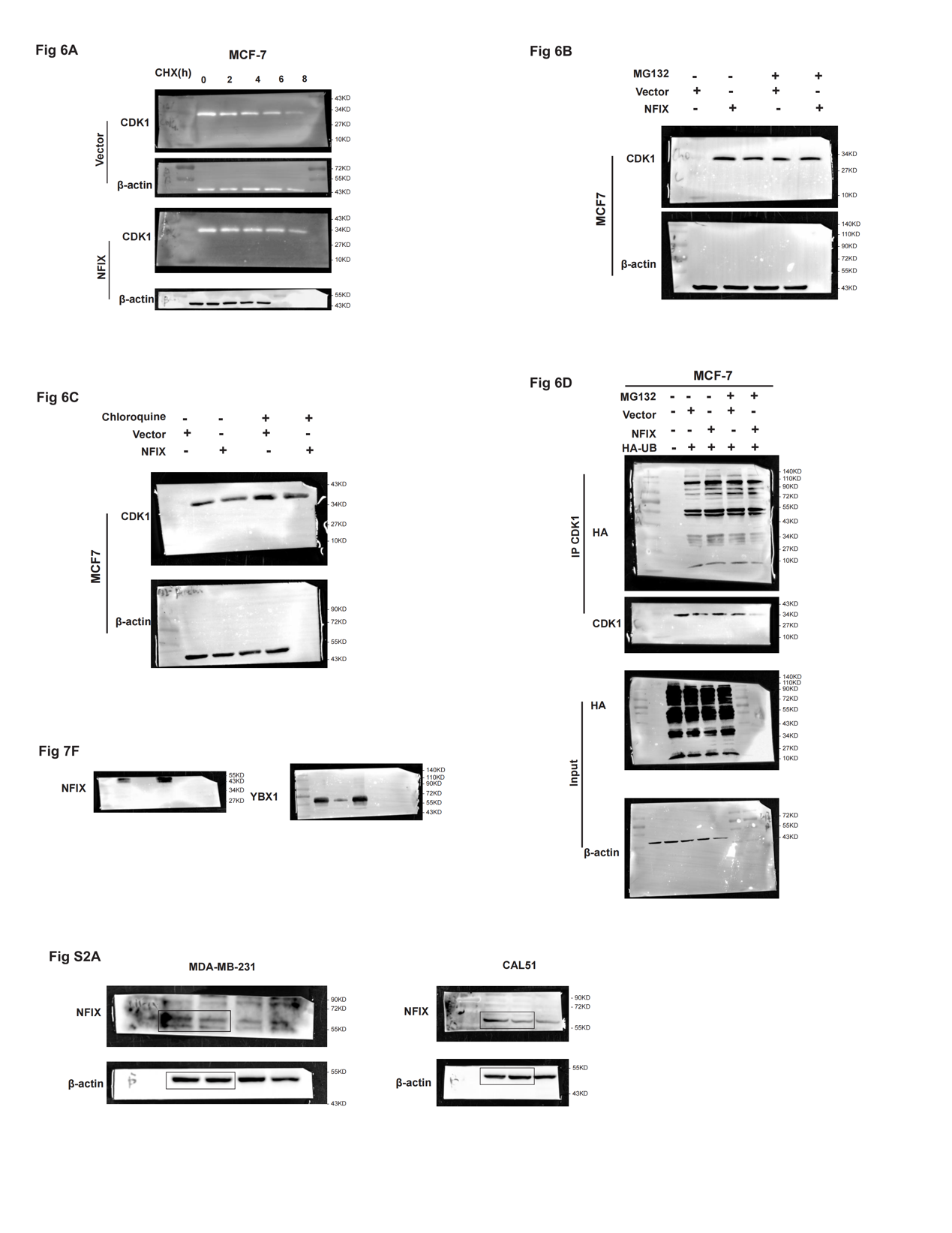

Supplement: Supplementary file 1 — Original western blot results [file 41420_2025_2361_MOESM1_ESM.docx]
