# Supplementary material for: NFIX suppresses breast cancer cell proliferation by delaying mitosis through downregulation of CDK1 expression
Source: Cell Death Discov. 2025 Feb 25;11:77. doi: 10.1038/s41420-025-02361-8 (PMC11861311; doi:10.1038/s41420-025-02361-8)
Supplement: Supplementary file 2 — Supplementary material [file 41420_2025_2361_MOESM2_ESM.docx]

**Supplemental Figures and Tables for**

**NFIX suppresses breast cancer cell proliferation by delaying mitotic process**

**through downregulating CDK1 expression**

**
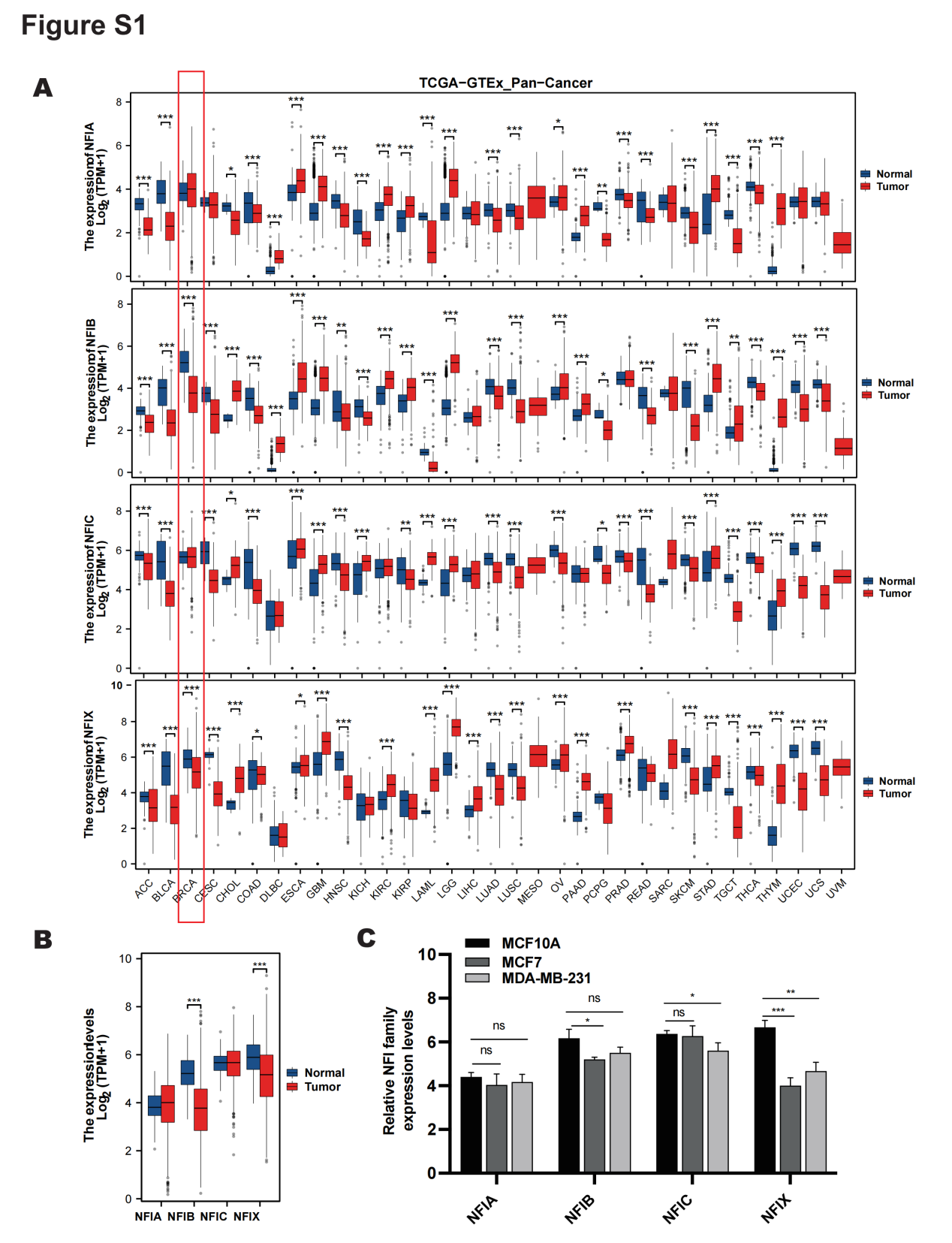
Fig.S1 The expression levels of NFI family in breast cancers.** (A) NFI family expression levels in multiple cancer types analyzed using the TCGA database. (B) NFIX expression levels in breast cancer analyzed using the TCGA database. (C) The NFI family expression levels in normal breast epithelial cells and in breast cancer cell lines.

**
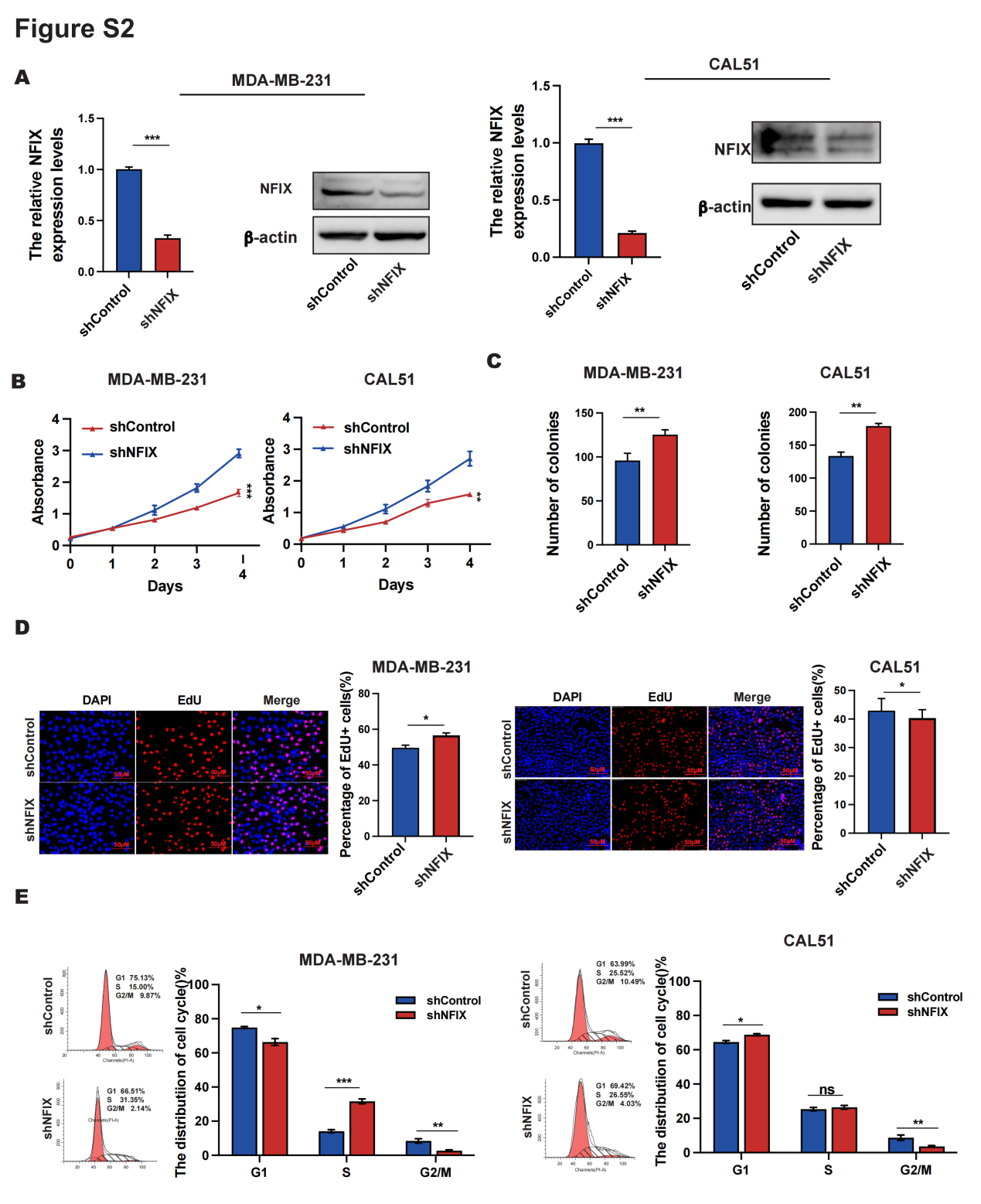
Fig.S2 NFIX deletion inhibited breast cancer cell proliferation.** (A) NFIX expression in stable NFIX‑depleted cells (231-shNFIX, CAL51-shNFIX) and control cells (231-shControl, CAL51-shControl) were determined by RT-qPCR and western blot analysis. (B–C) MTT (B) and colony-formation (C) assays were used to assess the proliferation of the cells described in (A). (D) EdU analysis of the proliferation of the cells described in (A). (E) Flow cytometry analysis of the cell cycle distribution of the cells described in (A). All experiments were repeated three times. Statistical significance was presented as *P < 0.05.

**
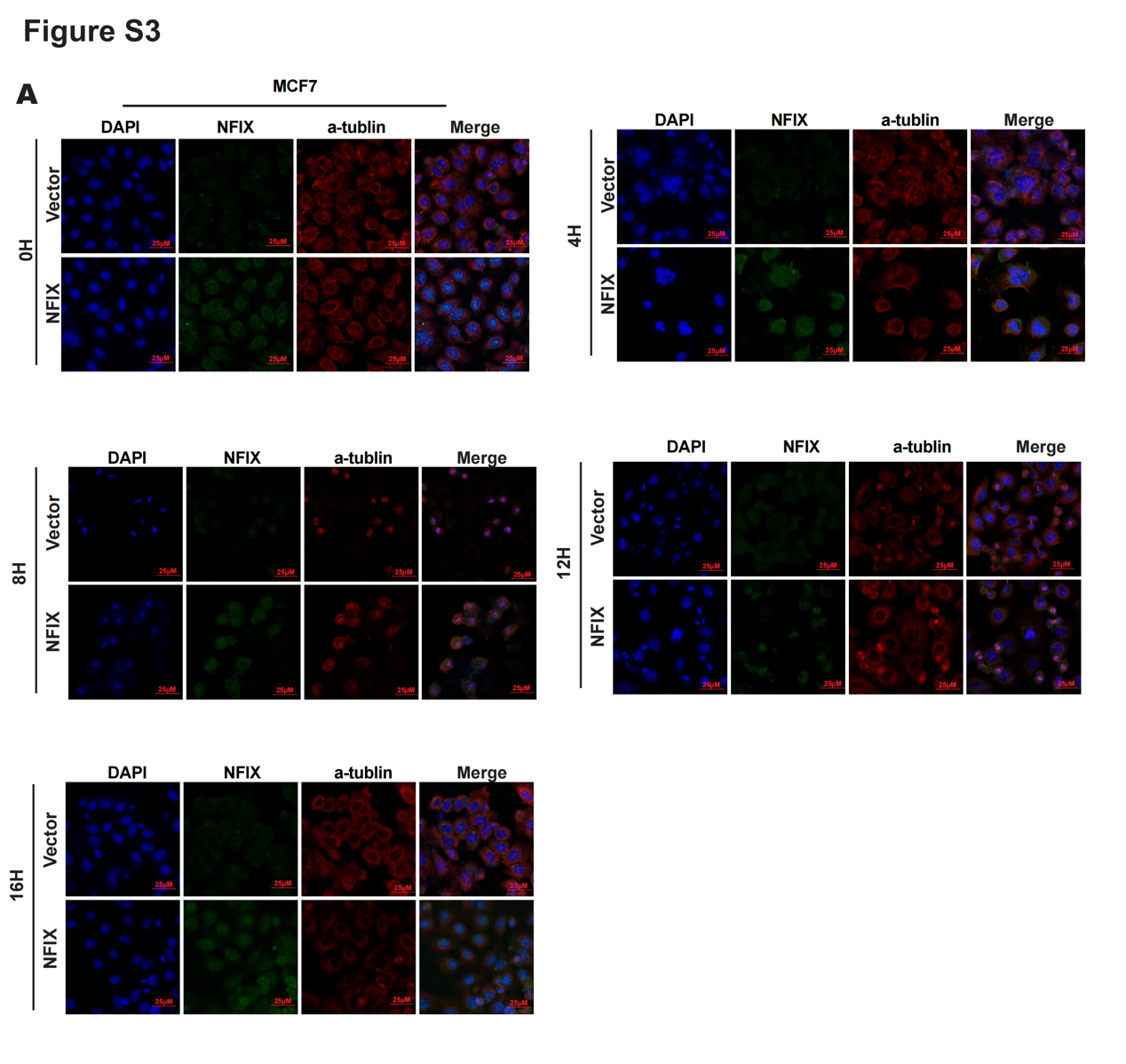
Fig.S3 Immunofluorescence staining of NFIX and Vector cells.** (A) The MCF7 cells were synchronized at the G1/S transition using a double-thymidine block and released for the indicated times.


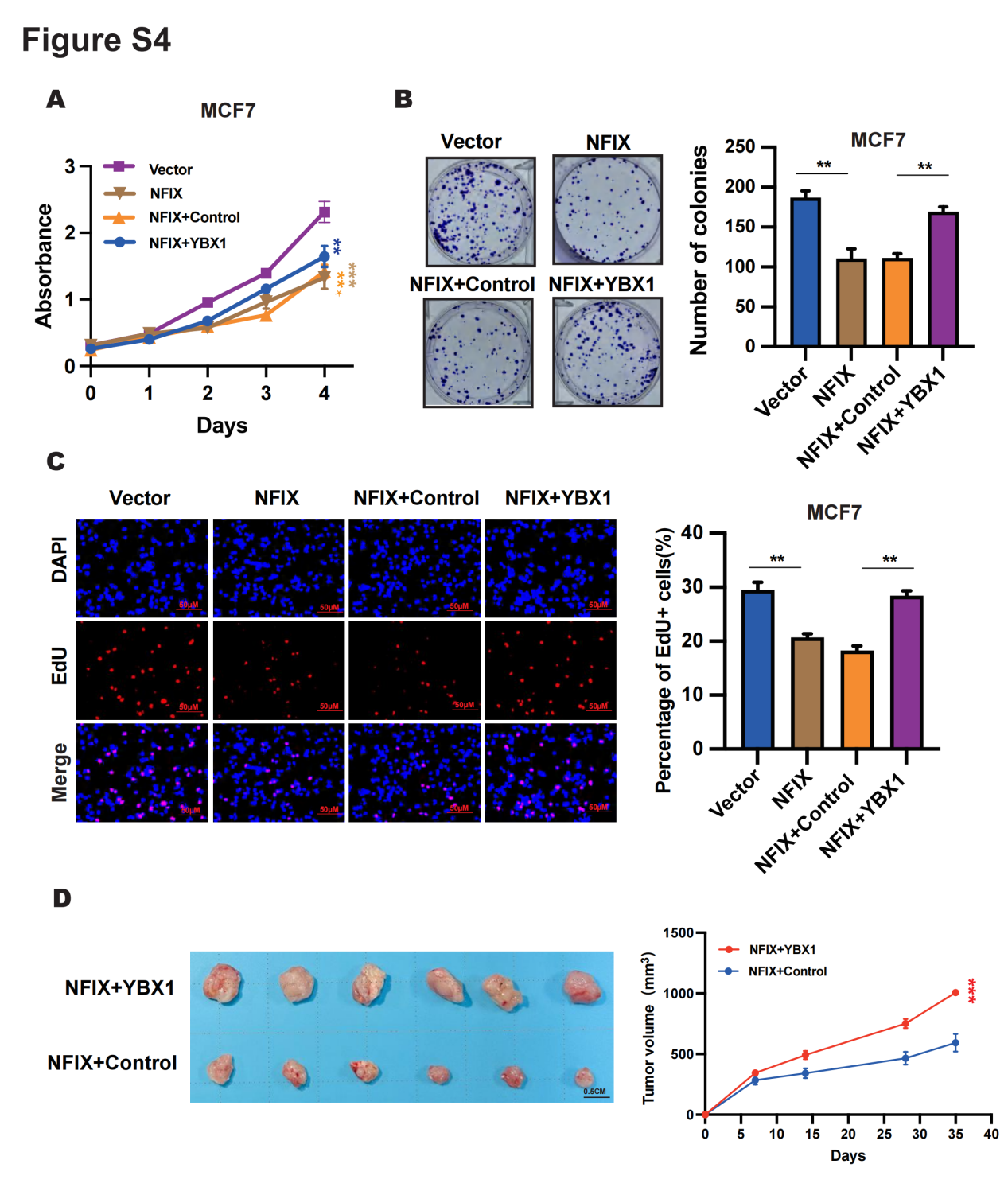


**Fig.S4 YBX1 rescues the effect of NFIX on breast cancer cell proliferation.** Cell growth promotion was determined by MTT (A), colony formation (B), and EdU (C) assays in MCF7 cells transfected with NFIX plasmid and YBX1 plasmid, as well as in control cells. (D)Tumor growth curves of subcutaneous xenograft tumors comprising NFIX- YBX1-overexpressing or NFIX+Control MCF7 cells at the indicated times.

**
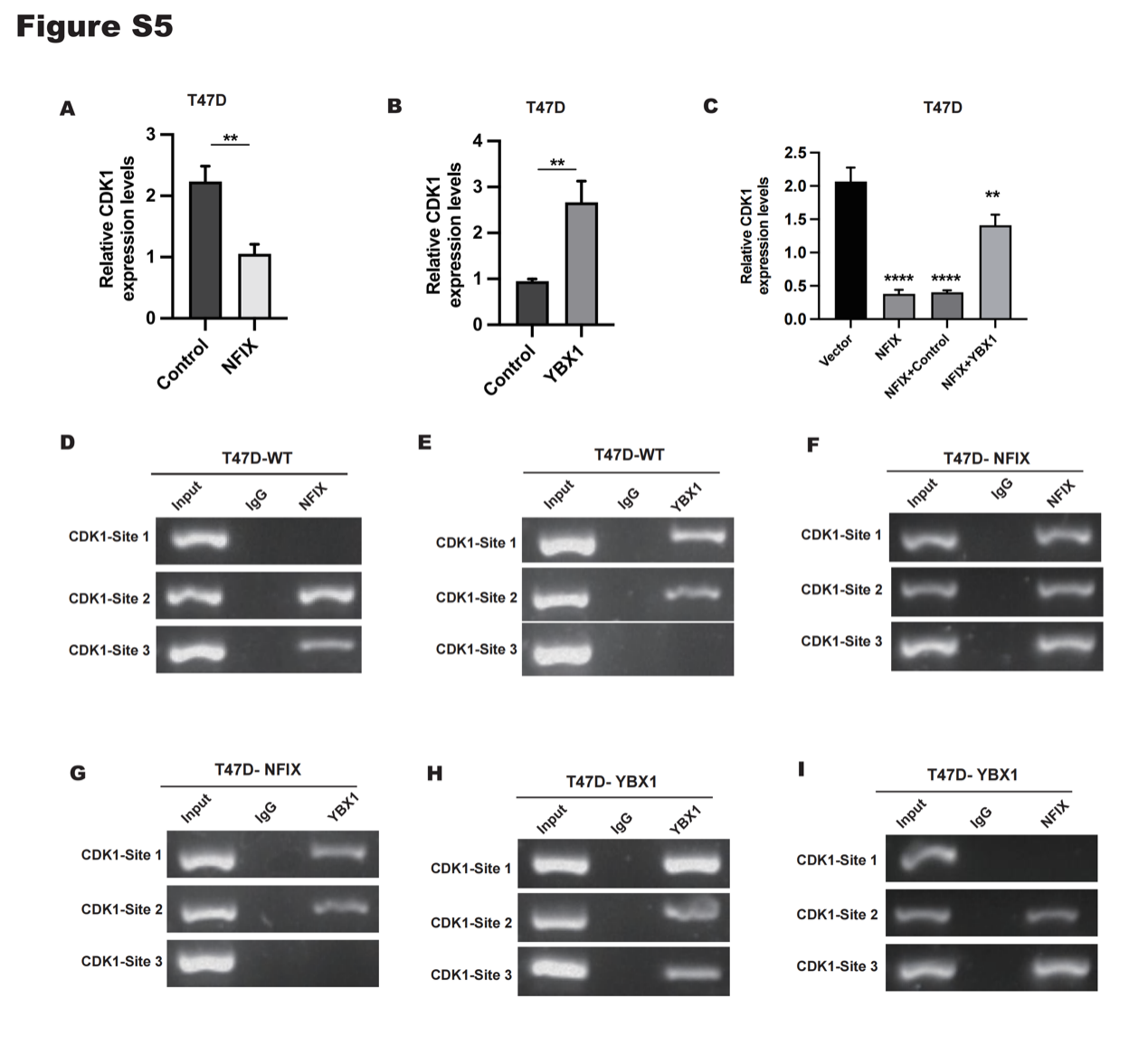
Fig.S5 NFIX transcriptionally inhibited CDK1 through YBX1 in T47D cells.** (A)The CDK1 mRNA expression level after transfection with the NFIX expression. Plasmid. (B)The CDK1 expression level after transfection with the YBX1 expression plasmid. (C)The expression levels of CDK1 in NFIX-overexpressing cells with or without transfection with the YBX1 plasmid and in the corresponding control cells were determined by qRT-PCR. (D) The binding of NFIX to site1, site2, and site3 according to ChIP analysis. (E) According to ChIP analysis, the binding of YBX1 to site1, site2, and site3 is in wild-type MCF7 cells. (F and G) The binding of NFIX and YBX1 to the CDK1 promoter region after transfection of the NFIX plasmid was verified by ChIP analysis. (H and I) The binding of NFIX and YBX1 to the CDK1 promoter region after transfection of the YBX1 plasmid was verified by ChIP analysis.

**
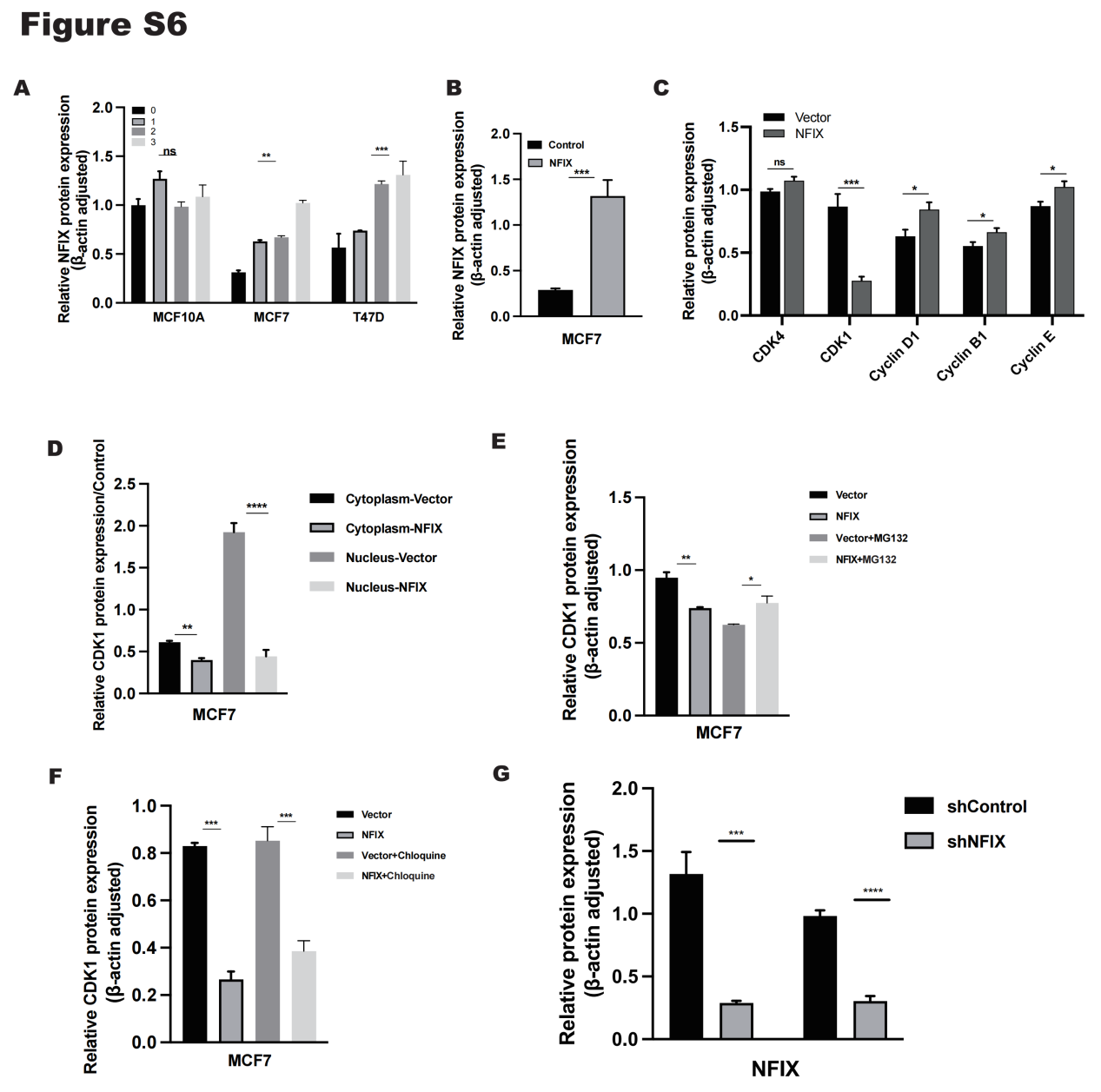
Fig.S6 The statistical analysis of immunoblots.**

The scale values of each single stripe were measured by Image J software and normalized by β-actin, including (A) Fig.2E, (B) Fig.3B, (C) Fig.5A, (D) Fig.5B, (E) Fig.6A, (F) Fig.6B, (G) Fig. S2A and Fig. S2B. All experiments were repeated three times. *p < 0.05, ***p < 0.01.

**Table S1.** Oligonucleotides used for RT-qPCR.

| **Name** | **Sequence (5’ to 3’)** |
| --- | --- |
| NFIX up  NFIX low | AGCAGTCGAGCCCGTATTTC  GTCCGATGCTGACAAACCG |
| CDK1 up  CDK1 low | AAACTACAGGTCAAGTGGTAGCC  TCCTGCATAAGCACATCCTGA |
| YBX-1 up  YBX-1 low | GGGGACAAGAAGGTCATCGC  CGAAGGTACTTCCTGGGGTTA |
| GAPDH up  GAPDH low | GGAGCGAGATCCCTCCAAAAT  GGCTGTTGTCATACTTCTCATGG |

**Table S2.** The Antibody information used in this research

|  | **Antibodies** | **Sourse** | **Cat.No.** |
| --- | --- | --- | --- |
| **Western blot** | NFIX | Novus | NBP2-15039 |
|  | P-21 | Proteintech | 10355-1-AP |
|  | P-16 | Proteintech | 10883-1-AP |
|  | Cyclin B1 | Santa Cruz | SC-752 |
|  | Cyclin D1 | Santa Cruz | SC-753 |
|  | β-actin | Cell Signaling Technology | 3700 |
|  | CDK1 | Santa Cruz | SC-54 |
|  | P-CDK1 | Abcam | ab275958 |
|  | YBX1 | Proteintech | 20339-1-AP |
|  | HA-tag | Immunoway | YM3003 |
|  | GAPDH | Sangene Biotech | KM9002 |
|  | 𝛼-tubulin | Proteintech | 80762-1-RR |
| **Co-IP** | NFIX | Novus | NBP2-15039 |
|  | CDK1 | Santa Cruz | SC-54 |
|  | YBX1 | Proteintech | 20339-1-AP |
|  | IgG | EMO milipore | 12-371 |
| **ChIP** | NFIX | Novus | NBP2-15039 |
|  | YBX1 | Proteintech | 20339-1-AP |
| **IHC** | NFIX | Novus | NBP2-15039 |
|  | Ki-67 | BD pharmingen | PAC047Ra01 |
|  | c-MYC | Proteintech | 10828-1-AP |

**Table S3.** Oligonucleotides used for ChIP and methylation-specific PCR.

| **Name** | **Sequence (5’ to 3’)** |
| --- | --- |
| CDK1 site 1 up  CDK1 site 1 low | CCTACCCTCAACACCTCCAG  AGAAGACCACAAGCTTCCACA |
| CDK1 site 2 up  CDK1 site 2 low | CGGTCTTTTGAGTTTTCCATTTCCT  CAAGCGCTCTCCTCCAGTC |
| CDK1 site 3 up  CDK1 site 3 low | GGTGTTTAGGTTGGGCTGGT  CTCTGTCTTCATTGCCATCCATCATATC |
| BSP up  BSP low | GTCATTTTCTGTCTTCTTGGGG  GGGATACCAGCAGCGTGTGTGT |
